# Pycnogonids associated with the giant lion´s-paw scallop *Nodipecten subnodosus* (Sowerby) in Ojo de Liebre Bay, Guerrero Negro, Baja California Sur, Mexico

**DOI:** 10.3897/zookeys.530.6064

**Published:** 2015-10-29

**Authors:** Angel de León-Espinosa, Jesus A. de León-González

**Affiliations:** 1 Universidad Autónoma de Nuevo León, Facultad de Ciencias Biológicas, Laboratorio de Biosistemática, Ap. Postal 5 “F”, San Nicolás de los Garza, Nuevo León. 66451 México

**Keywords:** Pycnogonida, new species, new records, Mexico, epifauna, *Nodipecten subnodosus*

## Abstract

Five species of epibenthic pycnogonids collected on the giant lion´s-paw scallop *Nodipecten subnodosus* are recorded. A new species of *Eurycyde*, *Eurycyde bamberi*, is described. Of the 19 species known in this genus; the new species is closest to *Eurycyde hispida* Kroyer, 1844 but differs from it in the absence of plumose spines and the shapes of the lateral process, first coxa, and ocular tubercle. The new species represents the third member of *Eurycyde* from the eastern Pacific in addition to *Eurycyde spinosa* Hilton, 1916 and *Eurycyde clitellaria* Stock, 1955. Besides *Eurycyde bamberi*, the following species were collected: *Nymphopsis duodorsospinosa* Hilton, 1942c; *Callipallene californiensis* (Hall, 1913); *Nymphon lituus* Child, 1979; and *Pycnogonum rickettsi* Schmitt, 1934. *Pycnogonum rickettsi* is recorded for first time from Mexican waters, as is *Nymphon lituus* from the western coast of Baja California Peninsula. Each of these four species are re-described and re-illustrated in order to fill in existing gaps in the literature of the region.

## Introduction

Pycnogonids are arthropods known as “sea spiders,” comprising a relatively small group of invertebrates that are distributed in all marine habitats from the intertidal zone to abyssal depths (Hedgpeth 1947, Arnaud and Bamber 1988, Genzano 2002, Cano-Sánchez and López-González 2007).

Pycnogonid studies in Mexico have been discontinuous and sporadic: Hilton (1942a) cited the first species from Mexico (*Nymphon pixellae* Scott, 1912), and later authors such as Hedgpeth (1948), Stock (1955), and Arnaud (1978) mentioned some pycnogonids from Mexican coasts. The most influential research for Mexico has been that of Child (1979), who reported 21 species from the Mexican Pacific. Munilla (2002) synthesized the information for Mexican littoral records, and found that 42 species were included in 17 genera and 6 families. In the present paper, we report the epibenthic pycnogonid specimens collected from the giant lion´s-paw scallop *Nodipecten subnodosus* (Sowerby).

## Methods

During a series of samplings made between 2012 and 2013 in Ojo de Liebre bay, Guerrero Negro, Baja California Sur, giant lion´s paw scallops were captured by scuba diving at depths not exceeding 10 meters in four fishing areas: El Datil (AD), El Chocolatero (AH), La Concha (AC) and El Zacatoso (AZ). Each scallop was collected individually in zip-lock bags to prevent loss of specimens associated with this clam. The biological material was transported in plastic containers to the station of the Centro de Investigaciones Biológicas del Noroeste in Guerrero Negro, the associated fauna were separated under a stereomicroscope, and placed in vials with 70% ethanol for later identification. Five pycnogonid species were found, and they were deposited in the Collection Carcinológica de la Facultad de Ciencias Biológicas, Universidad Autónoma de Nuevo León. For each species the material examined section is listed as follows: fishing area name, coordinates, catalog number (UANL-FCB-PYCNO), number of scallop, the number of specimens (in parentheses), and date.

## Systematic account

### Class Pycnogonida Latreille, 1810 Order Pantopoda Gerstäcker, 1863

#### Family Ammotheidae Dohrn, 1881

##### Genus *Eurycyde* Schiödte, 1857

###### Eurycyde bamberi sp. n.

Taxon classificationAnimaliaPantopodaAmmotheidae

http://zoobank.org/00096DAA-9488-42A6-8EE6-3BFC80552F22

Fig. 1

####### Material examined.

Holotype (1 male), Ojo de Liebre bay, Guerrero Negro, Baja California Sur, scallop fishing area: El Datil, 27°48'43"N, 114°15'06"W, (UANL-FCB-PYCNO-0031), AD-1, (1), 01/12/2012.

####### Description.

**Figure 1.**
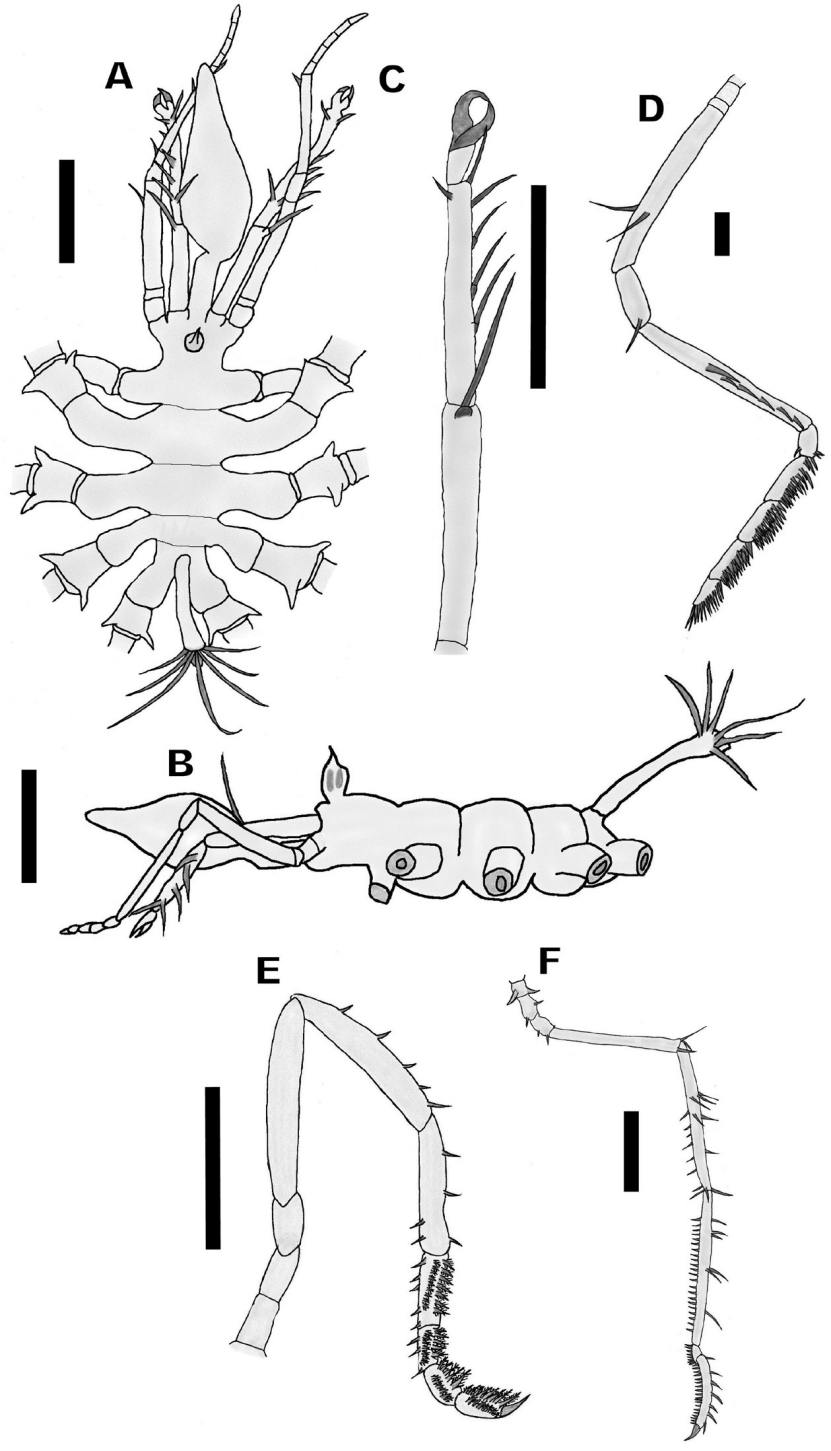
*Eurycyde bamberi* sp. n. Holotype, male (UANL-FCB-C-P000). **A** Trunk, dorsal view **B** Trunk, lateral view **C** Chelifore, lateral view **D** Palp, lateral view **E** Oviger, lateral view **F** Third leg, lateral view. Scale bars: 0.5 mm (**A–C, E–F**); 0.1 mm (**D**).

Proboscis articulated, proximal portion a cylindrical tube approximately one quarter of the total size, distal part pyriform, 3 smooth lips (Fig. 1A–B).

Chelifores with three segments, first scape segment slightly longer than second, with a long spine at the distal part directed forward, second scape segment with five ventrolateral spines and a smaller dorsal spine. Third segment approximately 1/3 the size of the second one, spineless, widening towards the distal part, ending in a smooth chela without auxiliary teeth (Fig. 1C).

Palp consists of ten segments, first one short, 0.05 mm long, second segment shorter than first one (1/3 its length), third segment approximately 0.55 mm long with two dorsal spines between the second and final third of the segment; the fourth segment smaller, 1/3 of the third one, with a spine on the distal end; fifth segment as long as the third one, with a line of lateral spines aligned forward starting in the second third of the segment and ending at the distal end; sixth segment small, half the size of the fourth, with three spines on the ventrodistal end, two on the ventral side and one at the dorsodistal end; seventh to ninth segments similar in both size and shape, with two rows of spines running along the entire ventral surface; tenth segment smaller than previous ones, with a row of spines on the ventral surface (Fig. 1D).

Small ocular tubercle, inserted at edge of cephalic segment, without lateral spines, twice as tall as its diameter capped with an inverted cone, with four pronounced eyes (Fig. 1B).

Trunk compact, spineless, suture lines slightly marked (Fig. 1A–B).

Lateral processes smooth, well-developed, longer than the width of the body and without spines or tubercles, separated by less than half of their own diameter.

With four long, slender walking leg pairs. Coxa I very short (0.1mm) with two thick dorsolateral tubercles, coxa II longer (0.17mm) with two short spines, one median dorsal and one ventrodistal, coxa III (0.14mm) slightly shorter than coxa II, with two short ventral spines, one median and one at the dorsodistal end. Femur smooth, armed with three long distal spines, one dorsal and two lateral. Tibiae I and II long, nearly subequal. Tibia I, armed with three dorsal and two mid-lateral spines, a long dorsodistal spine, seven ventral spines, smaller, in a row and two longer distal spines. Tibia II, with six long dorsal spines and a ventral row with 22 smaller setae. Tarsus, with a ventral row formed by eight setae. Propodus slightly curved, armed with seven dorsal spines and a ventral row of 19 sole spines. Thick claw, without auxiliary claws (Fig. 1F).

Oviger composed of 10 segments, first three short, segments 1 and 3 subequal, segment 2 slightly longer, fourth and fifth segments long and subequal, first to fourth segments without spines or setae, fifth segment with a ventral row of five moderately sized setae, sixth segment 2/3 the length of segment 5, with three ventral and two apical setae located dorsally, seventh to tenth segments smaller, with two rows of spines, the first row with the formula 7: 5: 5: 8, and the second row of spines similar in shape, but smaller than the first ones with the formula 9: 7: 6: 8. Last segment ends in a thick terminal claw (Fig. 1E). Eggs not observed.

Long cylindrical abdomen, extended at an angle of 45°, exceeding the length of the lateral processes and first coxae combined, of the fourth pair of legs; distal end of abdomen with 7 long thin spines, the rest smooth (Fig. 1B).

####### Standard measurements.

Proboscis 1.3 mm long, divided in two segments, proximal one of 0.35 mm long, distal segment 0.95 mm long, 0.35 mm wide.

Body 1.5 mm long from anterior end of cephalic segment to end of fourth lateral processes, 1 mm wide between second pair of lateral processes.

Leg 1 3.72 mm long from coxa I to the tip of main claw. Coxa I, 0.1 mm, coxa II, 0.17 mm, coxa III, 0.14 mm, femur 0.81 mm, tibia I, 0.89 mm, tibia II, 0.97 mm, tarsus, 0.08 mm, propodus 0.44 mm, claw 0.12 mm.

Oviger 2.45 mm long, first segment 0.09 mm, second 0.11 mm, third 0.09 mm, fourth 0.62 mm, fifth 0.56 mm, sixth 0.40 mm, seventh 0.2 mm, eighth 0.13 mm, ninth 0.12 mm, tenth 0.13 mm.

####### Distribution.

This species is known only from Ojo de Liebre bay, Guerrero Negro, Baja California Sur, Mexico.

####### Etymology.

Specific name is in honor of Roger Bamber for his great work on the knowledge of pycnogonids, who died recently on February 16, 2015.

####### Remarks.

*Eurycyde* is a relatively small genus. Until the present report, it was represented by 19 species and of these, only *Eurycyde spinosa* Hilton, 1916 and *Eurycyde clitellaria* Stock, 1955 have been previously recorded for the eastern Pacific. The first one was described from Laguna Beach, California, the second described from the Virgin Islands in the Caribbean Sea and later reported from Tenacatita Bay, Jalisco by Child (1979). This report is the third finding of a species of *Eurycyde* in the eastern Pacific. Table 1 shows important characteristic features of these *Eurycyde* species.

**Table 1.** Morphological characteristics of known *Eurycyde* species.

Species	Plumose spines	Lateral processes	Coxa I	Ocular tubercle shape	Type locality	Reference
*Eurycyde acanthopus* Stock, 1979	Present on 1^st^ and 2^nd^ tibia	With a dorsal spine	With two tall dorsal pointed spines	Tall and slender, without distal spines	Caracas, Venezuela	Stock (1979)
*Eurycyde antarctica* Child, 1987	Present on 1^st^ tibia	With a short dorsal tubercle inserted distally	Without associated structures	With broad base tapering to slender anterior tip, with four tiny slender tubercles	Adare Peninsula, Ross Sea	Child (1987)
*Eurycyde arctica* Child, 1995	Absent	With a short dorsal tubercle inserted distally	With two short latero-dorsal spines	Short, twice taller than its diameter	Amchitka Island, Aleutians	Child (1995)
*Eurycyde bamberi* sp.n.	Absent	Without associated structures	With two thick dorsolateral spines	Short, globose, distally pointed, twice taller than its diameter	Ojo de Liebre Bay, Baja California Sur, México	This work
*Eurycyde clitellaria* Stock, 1955	Present on 1^st^ and 2^nd^ tibia	With a dorsal spine	With two tall dorsal pointed spines	Tall and slender, with subdistal spines	Virgin Islands, Caribbean Sea	Muller and Krapp (2009)
*Eurycyde curvata* Child, 1979	Present on 1^st^ and 2^nd^ tibia	With a distal and lateral tubercles	With five tubercles inserted distally	Thin and tall, without distal spines	Cabo de la Vela, Colombia, Caribbean	Child (1979)
*Eurycyde depressa* Child, 1995	Absent	With two short latero-dorsal spines	With 3-4 short spines	Very low, as wide as tall	Semisopochnoi Island, Aleutians	Child (1995)
*Eurycyde diacantha* Stock, 1990	Present on distal end of femur, 1^st^ and 2^nd^ tibia	Without associated structures	With two heavy, almost triangular, pointed tubercles	Short, 2.5 times longer than its diameter	Cape Verde Islands	Stock (1990)
*Eurycyde flagella* Nakamura & Chullasorn, 2000	Present on chelifore, coxae I and II, and on ocular tubercle	First to third pairs of lateral processes with tall tubercles, fourth pair with low tubercles	With two long feathered spines on first two coxae	Tall, slender, four times longer than it´s diameter, with three feathered spines at tip	Puket Island, Thailand	Nakamura and Chullasorn (2000)
*Eurycyde gorda* Child, 1979	Present on coxa II, distal end of femur, 1^st^ and 2^nd^ tibia	Without associated structures	Without associated structures	Short, thick, with a distal circle of pointed spines	Galeta Island, Panamá, Caribbean	Child (1979)
*Eurycyde hispida* (Krøyer, 1844)	Present on legs and abdomen	With a setae inserted laterally	Without associated structures	Tall and thin	Greenland	Nakamura and Chullasorn (2000)
*Eurycyde longioculata* Muller, 1990	Absent	Without associated structures	With two thick dorsolateral spines, anterior one smaller than posterior one on first three pairs of coxae, last pair with equal size protuberances	Tall and thin, with more than five distal spines	Bora Bora, Society Islands, South Pacific	Müller (1990)
*Eurycyde longisetosa* Hilton, 1942	Present on distal end of femur, 1^st^ and 2^nd^ tibia	Without associated structures	With two latero-dorsal tubercles inserted distally	Short and slender, with two long and thin distal spines	Utria Harbor, Pacific of Colombia	Hilton (1942c)
*Eurycyde muricata* Child, 1995	Present along body	Present with 3-5 short spines	With two long lateral and one dorsal spines	Tall and slender, globose distally	Rat Islands Group, Aleutians	Child (1995)
*Eurycyde platyspina* Stock, 1992	Present on coxa II and III, distal end of femur, 1^st^ and 2^nd^ tibia	Present, a short dorsal tubercle inserted distally and some scattered spines	With two latero-dorsal tubercles inserted distally and two proximal spines	Short, three times taller than its diameter	North to Rio de Janeiro, Brasil	Stock (1992)
*Eurycyde raphiaster* Loman, 1912	Absent	With a dorsal tiny spine	With two protuberances, anterior one smaller than posterior one on first three pairs of coxae, last pair with equal size protuberances	With six long apical spines	Originally described from Monaco; type locality not specified, other records are from Caribbean Sea and Cape Verde Islands	Loman (1912); Hedgpeth (1948)
*Eurycyde sertula* Child, 1991	Present along body	First to third pairs of lateral processes with conical tubercles, fourth pair with smaller tubercles	First to third pairs of coxae with a small conical antero-distal tubercle, and a larger postero-distal tubercle armed with two long plumose spines	Short, 2.5 times longer than its diameter, with five long apical spines	Guam island, Philippine Sea	Child (1991)
*Eurycyde setosa* Child, 1988	Present along legs from coxae to tibia II	With a short rounded distal tubercle that decrease in size from anterior to posterior lateral processes	With two heavy, almost triangular, pointed tubercles, anterior one shorter than posterior one, both covered by tiny spines	Three times as long as maximum diameter, with seven spines inserted distally	Batan Island, Philippines	Child (1988)
*Eurycyde spinosa* Hilton, 1916	Absent	Without associated structures	With two thick dorsolateral spines and a single large spine	Short, conical	Laguna Beach, California	Hilton (1916)
*Eurycyde unispina* Stock, 1986	Present on 1^st^ and 2^nd^ tibia	Without associated structures	With a tall dorsal spur	Strongly pointed, with a distal spine	Straits of Florida	Stock (1986)

Based on the key proposed by Nakamura and Chullasorn (2000), this species is very close to *Eurycyde hispida* Kroyer, 1844, described from Greenland and whose type material has been lost; *Eurycyde hispida* has also occasionally been reported from the coasts of the north Atlantic. *Eurycyde hispida* has plumose spines on its legs and abdomen, lateral process with setae laterally, coxa I without a lateral tubercle, and a tall thin ocular tubercle. In contrast, the new species has simple spines on the abdomen and legs, lateral processes without tubercles or spines, coxa I with two dorsolateral spines, and the ocular tubercle short, narrowing towards the tip. Another closely related species is *Eurycyde spinosa* Hilton, 1916. These two species can be separated by the presence of a large posterior spine on each coxa I, and the ocular tubercle is conical with one large spine and several smaller spines in *Eurycyde spinosa*, while *Eurycyde bamberi* sp. n. does not have posterior spines on the first coxa, and the ocular tubercle is globose, distally pointed, without spines.

The following species are illustrated and described in full since their previous descriptions are quite outdated and in some cases, like *Nymphopsis duodorsospinosa*, very incomplete. This will help facilitate future identification of eastern Pacific pycnogonids as well as help to differentiate new species as they are collected and described from this region.

##### Genus *Nymphopsis* Haswell, 1885

###### Nymphopsis duodorsospinosa

Taxon classificationAnimaliaPantopodaAmmotheidae

Hilton, 1942c

Fig. 2

Nymphopsis duodorsospinosa Hilton 1942c: 303, pl. 45; Hilton 1943a: 98; Hedgpeth 1948: 250–252, fig. 40; Child and Hedgpeth 1971: 609; Kraeuter 1973: 496; Stock 1975: 978; Child 1979: 21.

####### Material examined.

Ojo de Liebre Bay, Guerrero Negro, Baja California Sur, scallop fishing area: La Concha, 27°50'35"N, 114°16'22"W, (UANL-FCB-PYCNO-0032), AC-3 (6♀, 1♂); (UANL-FCB-PYCNO-0033), AC-5 (1♀, 2♂); (UANL-FCB-PYCNO-0034), AC-6 (3♀, 1♂); (UANL-FCB-PYCNO-0035), AC-10 (9♂); (UANL-FCB-PYCNO-0036), AC-16(3♀, 2♂); (UANL-FCB-PYCNO-0037), AC-21 (1♀, 2♂); (UANL-FCB-PYCNO-0038), AC-22 (1♀, 4♂); (UANL-FCB-PYCNO-0039), AC-28 (1♂); (UANL-FCB-PYCNO-0040), AC-30 (2♀, 1♂); (UANL-FCB-PYCNO-0041), AC-31 (1♀), 01/10/2013; El Datil, 27°48'43"N, 114°15'06"W, (UANL-FCB-PYCNO-0042), AD-17 (1♀, 2♂), 01/12/2012; (UANL-FCB-PYCNO-0043), AD-(20) (2), 21/11/2013; El Zacatoso, 27°51'45"N, 114°12'19"W, (UANL-FCB-PYCNO-0044), AZ-2 (2♂); (UANL-FCB-PYCNO-0045), AZ-7 (1♀, 1♂); (UANL-FCB-PYCNO-0046), AZ-28 (1♀, 1♂) 01/09/2013.

####### Description.

**Figure 2.**
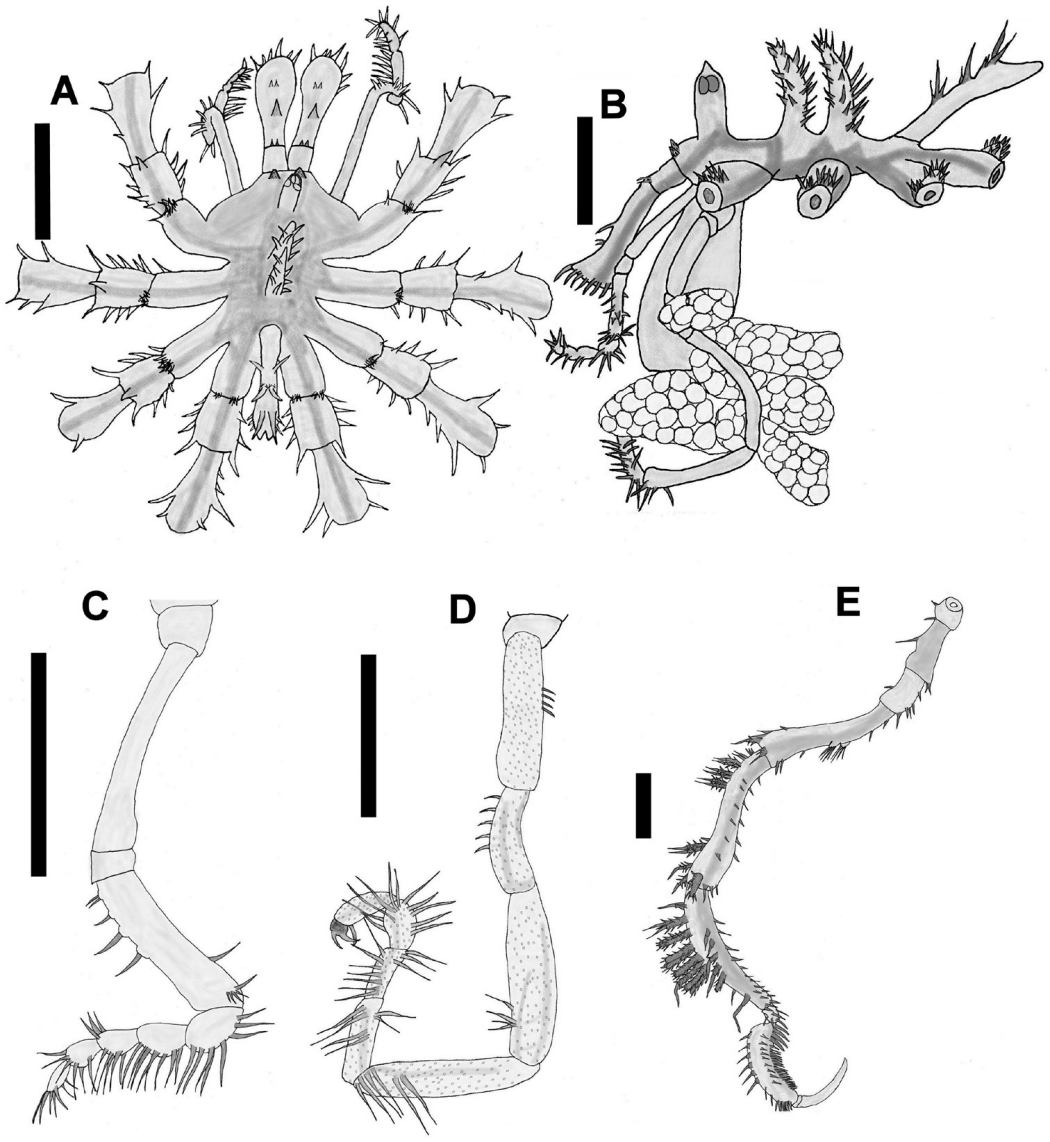
*Nymphopsis duodorsospinosa* Hilton, 1942. **A** Trunk, dorsal view **B** Trunk, lateral view **C** Palp, lateral view **D** Oviger, lateral view **E** Third leg, lateral view. Scale bars: 1 mm (**A–B, E**), 0.5 mm (**C–D**).

Proboscis cylindrical, vertical to body, with three smooth lips, narrow at the proximal portion, thicker toward the distal part, three times longer than wide (Fig. 2B).

Chelifore scape two-segmented, first one short, with two small setae on distal end, second one three times as long as 1^st^ segment, narrow for most of its length and widening at its distal end, with a long dorsal spine and two smaller distal setae located directly in front of the long spine. The widened distal end is encircled by a fringe of long setae. Chela small, retractable inside the wide end of the second scape segment (Fig. 2B).

Palp with nine segments, first and third short, second one longest, all these without setae. Segment four 2/3 length of segment 2, with a series of five dorsal setae, two isolated ventral setae, and a group of smaller basal setae; segments 5–9 with a row of long ventral setae; segments 7–8 each with a cluster of anterior distal setae (Fig. 2C).

A well-developed ocular tubercle inserted a little behind the anterior margin of the body, tall, cylindrical, ending in a conical apex, with four black eyes near the top (Fig. 2B).

Trunk slightly longer than wide, 3.1 mm long from anterior end of chelifore to distal end of abdomen, 2.1 mm wide between second pair of lateral processes, with spines on the dorsodistal end of the lateral processes. With two notable dorsal trunk tubercles covered with spines. No segmentation lines between body segments (Fig. 2A). Located just posterior to the insertion of the scape of the chelifore are two short dorsal tubercles surrounded by small spines (Fig. 2B).

Lateral processes well developed, longer than the width of the body, separated by a space equal to their own diameter, with one or two tufts of small setae on the dorso-distal end of each process. Lateral processes on legs 1–3 each have one or two additional longer spines at the dorso-distal end.

Legs adorned with numerous spines. Coxa I and III together, as long as coxa II, coxa I (0.3 mm) with a median dorsal spine (legs 1–3) and a row of lateral spines on each side, coxa II (0.67 mm) with a long dorsal spine inserted medially, and two ventrodistal spines on legs 1 and 2 (Fig. 2E); on legs 3 and 4, instead of those spines, there appears a ventrodistal tubercle adorned with seven pairs of lateral spines, present only in male specimens. The male gonopore is located on this tubercle, coxa III (0.48 mm) with a series of ventral setae and one longer dorsal seta. Femur and tibia I subequal, tibia II is slightly shorter. Femur with widely spaced setae on ventral side and distal end, tibia I with two rows of small setae on ventral side, and two dorsal groups of large complex spines, one group proximal and the other distal. Tibia II with one median dorsal row of large complex spines and one additional row of smaller complex spines off to one side. Tarsus small, curved, ventral surface with 5-6 spines. Propodus curved, five times longer than tarsus, with one median dorsal spine row and two lateral spine rows. At the dorsodistal end, there is a cluster of smaller spines. The ventral surface of the propodus has four large thick heel spines and a row of smaller sole spines. Long curved terminal claw, 85% the length of the propodus, auxiliary claws absent. (Fig. 2E).

Oviger formed by ten segments, first one very short, second, fourth, and fifth longest, nearly subequal, third segment is 2/3 the length of segment 2 and curved, armed with a dorsal row of setae, fourth with a small cluster of dorsodistal setae, fifth with two long lateral spines and a ventral cluster of long spines at the distal end. Segment six with two lateral groups of two spines each, and a dorso-distal group of two smaller spines. Segment seven with a lateral row of seven long spines, and a dorso-distal row of three spines; segment eight with a row of five dorsal spines, a row of four lateral spines and two ventral spines; segment nine longer than seven, with a dorso-ventral hook-like spine. Segment ten very small, with two hook-like terminal spines (Fig. 2D).

Long slightly curved abdomen, directed posteriorly at an angle less than 45°, with three pairs of dorsal spines, each one with small setae at the base (Fig. 2B).

####### Standard measurements.

Proboscis 1.5 mm long, 0.76 mm wide.

Body 1.45 mm long from anterior end of cephalic segment to end of 4th lateral processes, 2.57 mm wide between second pair of lateral processes.

Leg 1 7.98 mm long from coxa I to the tip of main claw. Coxa I, 0.3 mm, coxa II, 0.67 mm, coxa III, 0.48 mm, femur 1.58 mm, tibia I, 1.58 mm, tibia II, 1.5 mm, tarsus, 0.3 mm, propodus 0.8 mm, claw 0.68 mm.

Oviger 2.71 mm long, first segment 0.06 mm, second 0.49 mm, third 0.32 mm, fourth 0.53 mm, fifth 0.56 mm, sixth 0.24 mm, seventh 0.19 mm, eighth 0.14 mm, ninth 0.18 mm, and tenth 0.04 mm.

####### Distribution.

The type locality of *Nymphopsis duodorsospinosa* is San Francisquito Bay, Gulf of California (Hilton 1942c); Hilton (1943a) recorded this species from San Francisco Bay to Lower California, including several localities in the Gulf of California and also from the Galapagos. Hedgpeth (1948) cited this species from South Carolina and Florida, Child and Hedgpeth (1971) listed this species from the Galapagos. Child (1979) recorded this species from western Mexico and both coasts of Panama.

####### Remarks.

Hilton (1942c) noted that *Nymphopsis duodorsospinosa* is close to *Nymphopsis spinosissima* (Hall, 1912); however, these can be differentiated by the number of dorsal tubercles (two and three respectively), differences in the chelifore and chelifore scape, spination on the abdomen, lateral processes, and legs, propodal heel and sole spines, and size and shape of the eye tubercle. According to Hedgpeth (1948), the oviger of *Nymphopsis duodorsospinosa* is formed by ten segments, not nine as described by Hilton (1942c). Furthermore, the fifth segment in *Nymphopsis duodorsospinosa* is larger with a basal group of spines, not short and covered on all sides with small “hairs” as in *Nymphopsis spinosissima*.

#### Family Callipallenidae Flynn, 1929

##### Genus *Callipallene* Flynn, 1929

###### Callipallene californiensis

Taxon classificationAnimaliaPantopodaCallipallenidae

(Hall, 1913)

Fig. 3

Pallene californiensis Hall 1913: 133, Pl. 4, figs 9–13; Hilton 1915a: 67; 1915b: 204; 1916: 465, fig. 6; 1920: 93.
Callipallene californiensis : Hilton 1942b: 281, pl. 36; 1942c: 38.
Callipallene solicitatus Child 1979: 44–46, fig. 15.

####### Material examined.

Ojo de Liebre Bay, Guerrero Negro, Baja California Sur, scallop fishing area: La Concha, 27°50'35"N, 114°16'22"W, (UANL-FCB-PYCNO-0047), AC-2, (1♂), 01/12/2012.

####### Description.

**Figure 3.**
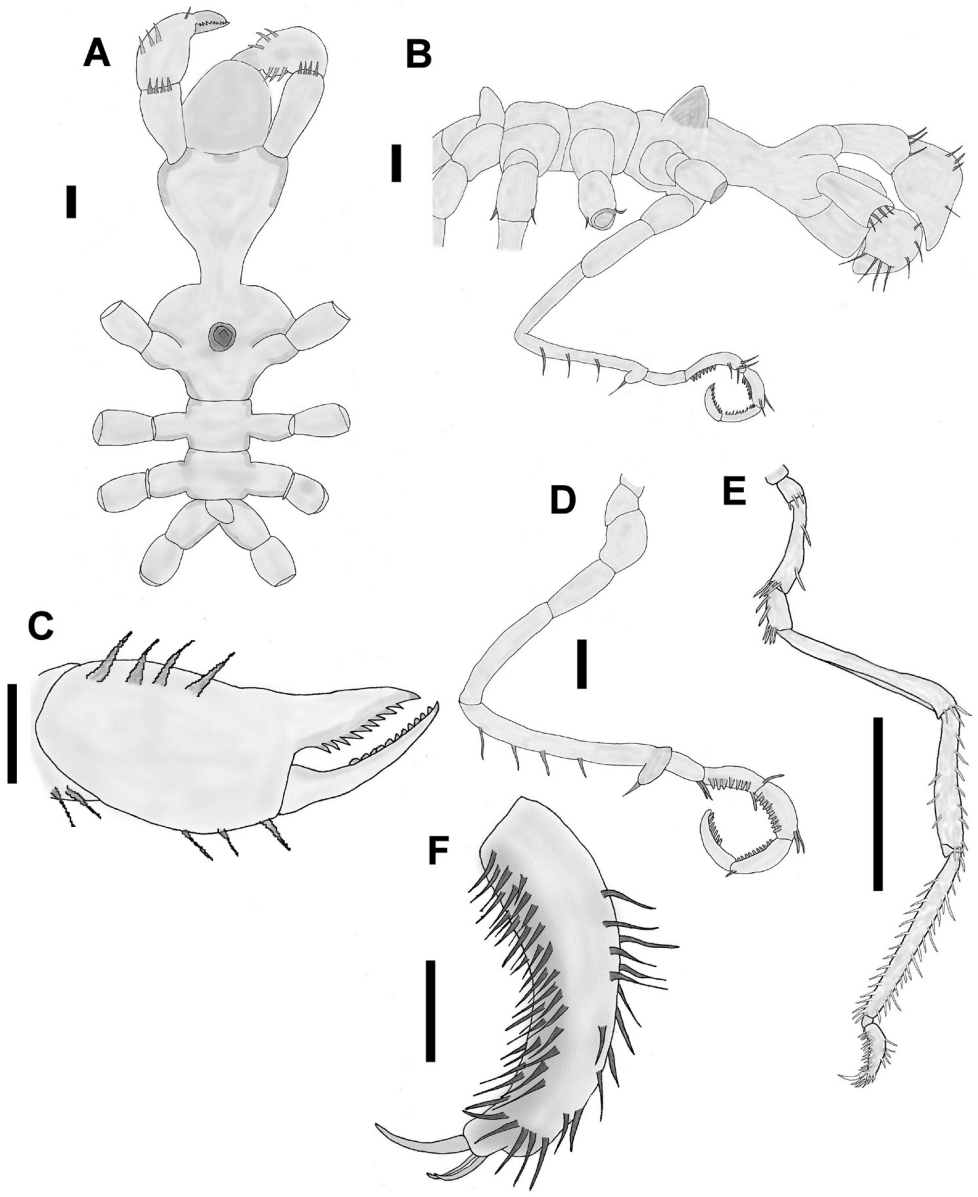
*Callipallene californiensis* (Hall, 1913). **A** Trunk, dorsal view **B** Trunk, lateral view **C** Chela, lateral view **D** Oviger, lateral view **E** Third leg, lateral view **F** Propodus, lateral view. Scale bars: 0.1 mm (**A–D, F**), 1 mm (**E**).

Proboscis short and rounded distally (Fig. 3A), with three smooth lips.

Chelifores with two segments: scape one-segmented, short, with a distal row of short spines. Chela large, chelate, with two dorsal and lateral rows of three spines each. The inferior chela finger is thin, articulated, with eleven teeth, extending beyond the distal portion of the upper finger, armed with nine teeth (Fig. 3C).

Palps absent. Ocular tubercle conical and apparently eyeless, located on cephalic segment just forward of the first pair of lateral processes (Fig. 3B).

Trunk short, robust, anterior corners of the first body segment rounded. Slender neck basally, almost as thick as its length in the distal part (Fig. 3A). Distinct segmentation lines between body segments.

Lateral processes without accessory structures, first and second pairs separated approximately by twice their diameters, second to fourth pairs separated approximately by their own diameter (Fig. 3A, B).

Oviger consists of ten segments, first three short, fourth segment as long as the second and third ones together. Fifth segment longest, with an apophysis at the distal end and a row of four long spines on the ventral surface. Apophysis has several long ventral setae. Sixth segment short, with a cluster of long setae at the ventrodistal end. Segments seven to ten (strigilis) each have a single ventral row of denticulate oviger spines with the formula 7: 8: 10: 7, and long setae are present on the dorsodistal end of segments seven, eight, and nine (Fig. 3D). Terminal oviger claw absent.

Legs consisting of eight segments (Fig. 3E). Coxa I very short, with a row of setae along the distalmost edge of the segment. Coxa II, longest of the three coxae, with two spines on the dorsal side and a cluster of long spines at the distalmost ventral edge of the segment. Coxa III, slightly longer than coxa I, with two spines on the ventral surface and a cluster of long spines at the distalmost ventral edge of the segment. Femur narrow in its proximal part, broadening at the distal end, with a row of long spines at the dorsodistal end. Tibia I with a row of long spines covering the entire dorsal surface and a short row of spines covering the second half of the ventral surface. Tibia II with a row of long spines running nearly the entire length of the ventral surface and a row of spines running the entire length of the dorsal surface, in the following pattern: 2–4 short spines then a long spine (twice as long as the short spines), repeated several times. Tarsus short, curved, about half the length of coxa I, without spines. Propodus with a scattered row of long spines on the dorsal surface and a row of long spines at the distalmost end. There are several rows of long heel and sole spines on the ventral surface, a thick terminal claw less than ½ (0.41) the length of the propodus, and two thin auxiliary claws, ½ the length of the terminal claw (Fig. 3F).

Abdomen short, conical, as long as its diameter, located above the fourth segment of the body, its front end marking the separation between the third and fourth segments (Fig. 3A–B).

####### Standard measurements.

Proboscis 0.3 mm long, 0.29 mm wide.

Body 0.7 mm long from anterior end of cephalic segment to end of fourth lateral processes, 0.43 mm wide between second pair of lateral processes.

Leg 1 4.39 mm long from coxa I to the tip of main claw. Coxa I, 0.15 mm, coxa II, 0.54 mm, coxa III, 0.23 mm, femur 1.08 mm, tibia I, 0.78 mm, tibia II, 1.08 mm, tarsus, 0.05 mm, propodus 0.34 mm, claw 0.14 mm.

Oviger 1.5 mm long, first segment 0.05 mm, second 0.1 mm, third 0.14 mm, fourth 0.25 mm, fifth 0.37 mm, sixth 0.11 mm, seventh 0.12 mm, eighth 0.11 mm, ninth 0.13 mm, tenth 0.12 mm.

####### Distribution.

Laguna Beach, California, La Paz Bay, Gulf of California, and Pacific coast of Panama.

####### Remarks.

*Callipallene californiensis* (Hall, 1913) had been reported rarely and appeared to be restricted to California. Hilton (1942c) re-described the species and provided an illustration of a complete specimen. Later, Child (1979) described *Callipallene solicitatus* from La Paz Bay, Gulf of California and the Pacific coast of Panama, providing a complete description and illustrations. Child (1987) reviewed the types of Hall and commented upon previous reports of this species, as well as designating *Callipallene solicitatus* a junior synonym of *Callipallene californiensis*. Our specimen agrees with the original description of *Callipallene californiensis*, and is located within its range of distribution (California to Panama). However, one character varies significantly, the proportion of the main claw is 75% the length of the propodus in *Callipallene californiensis*, whereas in our specimen it is 41%; otherwise, all other characters are similar.

#### Family Nymphonidae Wilson, 1878

##### Genus *Nymphon* Fabricius, 1794

###### Nymphon lituus

Taxon classificationAnimaliaPantopodaNymphonidae

Child, 1979

Fig. 4

Nymphon lituus Child 1979: 38–40, fig. 13.

####### Material examined.

Ojo de Liebre Bay, Guerrero Negro, Baja California Sur, scallop fishing areas: Chocolatero, 27°53'04"N, 114°15'06"W, (UANL-FCB-PYCNO-0048), AH-28, (1♂), 01/10/2012; El Datil, 27°48'43"N, 114°15'06"W, (UANL-FCB-PYCNO-0049), AD-8, (1♂, 1♀), 01/12/2012; La Concha, 27°50'35"N, 114°16'22"W, (UANL-FCB-PYCNO-0050), AC-11, (1♂), 01/12/2012; (UANL-FCB-PYCNO-0051), AC-14, (1♂); (UANL-FCB-PYCNO-0052), AC-15, (1♀), 01/10/2013; (UANL-FCB-PYCNO-0053), AC-10, (1♂), 22/11/2013.

####### Description.

**Figure 4.**
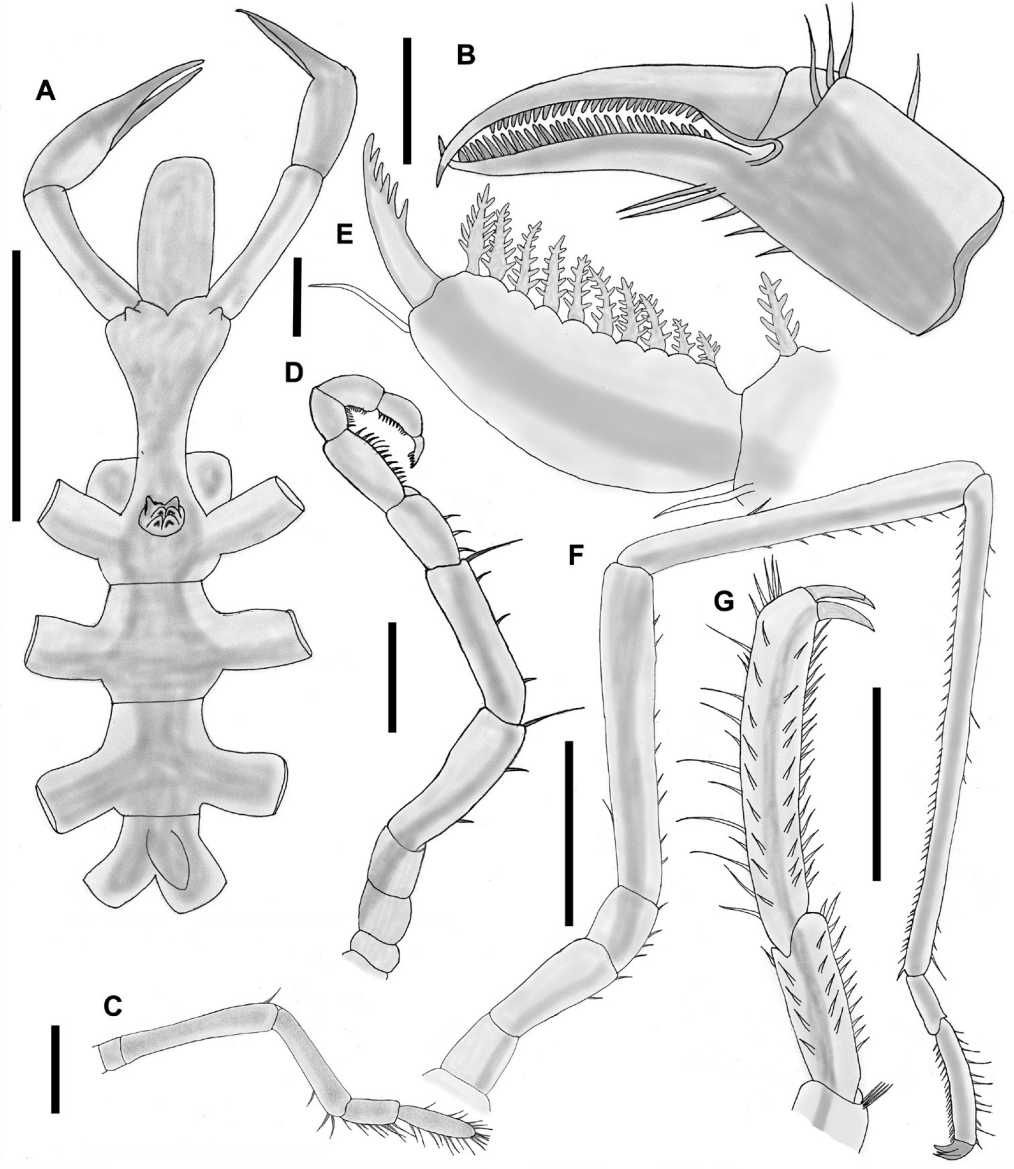
*Nymphon lituus* Child, 1979. **A** Trunk, dorsal view **B** Chela, lateral view **C** Palp, lateral view **D** Oviger, lateral view **E** Oviger, terminal end, lateral view **F** Third leg, lateral view **G** Propodus, lateral view. Scale bars: 1 mm (**A, F**), 100 µm (**B**), 0.5 mm (**C–D, G**), 50 µm (**E**).

Proboscis cylindrical, longer than wide, horizontal to body (Fig. 4A), with three smooth lips.

Chelifore with two segments, the scape cylindrical. Fingers of the chela slender, longer than the basal part, which is inflated and rectangular, with a single median dorsal spine and 3 large dorsal setae in a row at the distal end, next to the articulation with the movable finger; and 2 long and 3 shorter setae on the ventral surface. The fixed finger has 29–30 slender chela teeth. Upper movable finger without setae, armed with 25 small teeth. The tips of the fingers slightly curved, overlapping distally (Fig. 4B).

Palps of five segments. First segment, very short. Second segment longest, with one large dorsodistal seta. Third segment is 2/3 the length of segment two with three ventral isolated setae. Fourth segment twice as long as segment one, with a ventral row of eight setae. Fifth segment 1.5 times as long as fourth segment, with two parallel rows of nine and seven long ventral setae, terminal end with a cluster of four setae (Fig. 4C).

Ocular tubercle inserted between the first pair of lateral processes, cone-shaped, with two small projections in the form of papillae, with two pairs of eyes (Fig. 4A).

Trunk slender, segmented. Neck 4.5 times longer than its width, smaller, cylindrical, widening in the form of a calyx, with a pair of conical, short anterior projections (Fig. 4A).

Lateral processes between first and second pairs separated slightly by their own diameters, second and third pairs separated by 1.5 times their diameters, and third and fourth pairs separated by less than their own diameters (Fig. 4A).

Legs long and slender (Fig. 4F, G). Coxa I short, without setae. Coxa II two times longer than coxa I, with two ventral anterior setae. Coxa III slightly longer than coxa I, with a row of four small ventral setae. Femur long, with one dorsal seta and scattered ventral setae. Tibia I with a single long median dorsal seta, and a ventral row of 9 smaller setae. Tibia II with a row of 45–50 small ventral setae, and 8–9 dorsal setae. Tarsus half as long as propodus with 10–11 ventral setae, a lateral row of six setae and a dorsal row of four setae. Propodus two times longer than tarsus, slightly curved, with a row of 19–20 sole spines, a median lateral row of ten spines, and two dorsal rows of 18–20 spines each, one row composed of short and the other of long spines. Main claw short, less than ¼ the length of the propodus, auxiliary claws nearly as long as main claw (Fig. 4G).

Oviger (Fig. 4D, E) inserted in the distal half of the first lateral process. First three segments short, first segment is half the size of the second one, second and third segments are subequal. Fourth and fifth segments longest, subequal. Fourth segment with two short ventral setae, and one long ventrodistal spine, fifth segment with a ventral row of three setae and one long ventrodistal spine. Sixth segment as long as the first and second together, with a ventral row of three setae. Last four segments armed with compound ovigerous leg spines, each with the following formula: 13: 9: 8: 10. Compound spines with 3–6 pairs of lateral teeth depending upon the size of the spine. Terminal claw long, curved, with six teeth (Fig. 4E).

Female gonopores oval in shape, present on ventrodistal end of coxa II of all leg pairs. These were observed on only two specimens (AD-8 and AC-15). All other specimens (males) without readily visible gonopores.

Abdomen as long as lateral processes of 4^th^ pair of legs, elevated from the body at an angle of about 30° (Fig. 4A).

####### Standard measurements.

Proboscis 0.57 mm long, 0.28 mm wide.

Body 1.43 mm long from anterior end of cephalic segment to end of 4th lateral processes, 1.0 mm wide between second pair of lateral processes.

Leg 1 9.35 mm long from coxa I to the tip of main claw. Coxa I, 0.37 mm, coxa II, 0.71 mm, coxa III, 0.41 mm, femur 1.84 mm, tibia I, 2.02 mm, tibia II, 2.78 mm, tarsus, 0.35 mm, propodus 0.71 mm, claw 0.16 mm.

Oviger 3.19 mm long, first segment 0.78 mm, second 0.81 mm, third 0.38 mm, fourth 0.44 mm, fifth 0.19 mm, sixth 0.22 mm, seventh 0.38 mm, eighth 0.24 mm, ninth 0.24 mm, tenth 0.21 mm.

####### Distribution.

This species is known from Gulf of California and Panama: specimens from Gulf of California were taken on floating *Sargassum* around Puerto Peñasco, Sonora, and in Panama City, among hydroids and bryozoans (Child 1979, p. 40). With this record, the distribution of *Nymphon lituus* is extended to the western coast of Baja California Peninsula.

####### Remarks.

The genus *Nymphon* includes 277 valid species (Bamber et al. 2015). Despite the great diversity of the genus, only ten species are known from the eastern Pacific: *Nymphon aculeatum* Child, 1994 from San Clemente Basin, California; *Nymphon apheles* Child, 1979 from Panama; *Nymphon duospinum* (Hilton, 1942) from Alaska; *Nymphon heterodenticulatum* Hedgpeth, 1941 from Santa Catalina Island, southern California; *Nymphon hirsutum* Child, 1995 from the Bering Sea; *Nymphon lituus* Child, 1979 from the Gulf of California and Panama; *Nymphon longicollum* Hoek, 1881 from Chile (also from New Zealand and Auckland Islands); *Nymphon pixellae* Scott, 1912 from Vancouver, Canada (also from California and Japan); *Nymphon similis* Child, 1992 from Ecuador; and *Nymphon stipulum* Child, 1990 from southern California. *Nymphon lituus* is a species known only from its original description; the specimens found in this study vary slightly in the number of compound ovigerous leg spines, with the formula 13: 9: 8: 10, in contrast to the original description of *Nymphon lituus* with the formula 15: 10: 10: 11. The other features do not present major variations.

#### Family Pycnogonidae Wilson, 1878

##### Genus *Pycnogonum* Brünnich, 1764

###### Pycnogonum rickettsi

Taxon classificationAnimaliaPantopodaPycnogonidae

Schmitt, 1934

Fig. 5

Pycnogonum rickettsi Schmitt 1934: 62, Fig. 1 A-D.
Pycnogonum rickettsi . Child and Hedgpeth 2007: 665; Hilton 1943b: 19; Hedgpeth 1975: 41a7, 424; pl. 99, fig. 3; Hedgpeth and Haderlie 1980:638, fig. 27.2.

####### Material examined.

Ojo de Liebre Bay, Guerrero Negro, Baja California Sur, scallop fishing area: La Concha, 27°50'35"N, 114°16'22"W, (UANL-FCB-PYCNO-0054), AC-3 (1♀), 01/12/2012.

####### Description.

**Figure 5.**
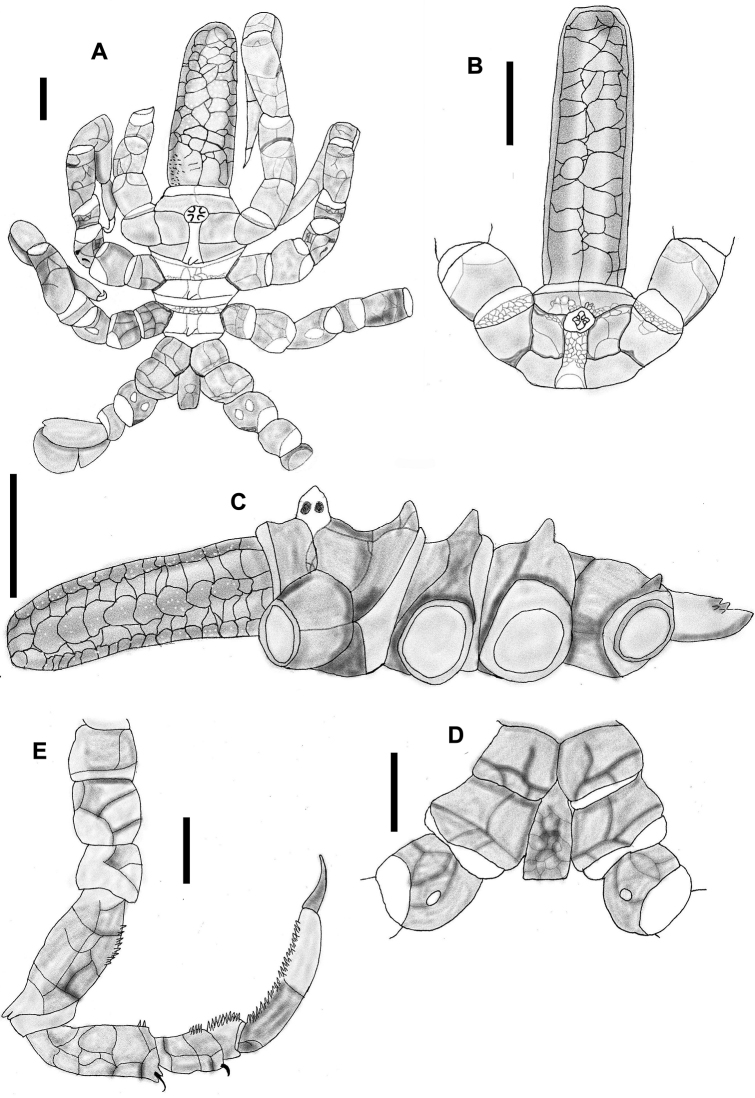
*Pycnogonum rickettsi* Schmitt, 1934. **A** Trunk, dorsal view **B** Trunk, dorsal view detail anterior end **C** Trunk, lateral view **D** Trunk, dorsal view, detail posterior end **E** Third leg, lateral view. Scale bars 1 mm (**A–C**), 0.5 mm (**D–E**).

Proboscis robust, longer than wide, slightly down-curved, articular membrane at base of proboscis narrow (Fig. 5A–C).

Ocular tubercle high, bell-shaped, with two pairs of large strongly pigmented eyes (Fig. 5C).

Trunk robust, segmented, integument granular (Fig. 5A, C), with reticulations evident on dorsal and ventral surfaces. Fully segmented, first three trunk segments armed with a high dorsal ridge at posterior end of each body segment.

Lateral processes separated by approximately one third of their width, all as long as wide, those of the first segment are directed forward, the second and third lateral processes are directed straight out and the fourth ones point backwards (Fig. 5A).

Legs: Coxae I and II subequal, coxa III shortest, articular membrane between segments wide. Femur is the longest segment, with two conical projections on dorsodistal end and a group of isolated setae on ventral surface. Tibia I is slightly shorter than femur and is nearly twice the length of tibia II, with two dorsodorsal conical projections, similar to those found on the femur, with a strong recurved spine between the conical projections, and isolated setae on the ventral surface; tibia II short, with a slight dorsal depression mid-segment, a strong distal recurved spine, and a small group of ventral setae placed in 3–4 regular rows. Tarsus short, with 6–7 rows of setae that almost completely cover the ventral surface. Propodus nearly as long as femur, with four rows of sole spines. Claw approximately 50% of propodus length, auxiliary claws absent (Fig. 5E).

Chelifores: absent

Palps: absent

Oviger: absent.

Female gonopore evident, oval, well-defined, situated on dorso-lateral surface of coxa II of fourth pair of legs (Fig. 5D).

Abdomen 0.8 mm long, smooth, cylindrical, reaching distal margin of coxa I on fourth pair of legs, with four small spines on middle dorsal area (Fig. 5C), posterior end truncate (Fig. 5A, D), and anus terminal.

####### Standard measurements.

Proboscis 2.2 mm long, 0.9 mm wide.

Body 2.08 mm long from anterior end of cephalic segment to end of fourth lateral processes, 1.91 mm wide between second pair of lateral processes.

Leg 1 6.0 mm long from coxa I to the tip of main claw. Coxa I, 0.5 mm, coxa II, 0.6 mm, coxa III, 0.3 mm, femur 1.2 mm, tibia I, 1.1 mm, tibia II, 0.6 mm, tarsus, 0.2 mm, propodus 1.0 mm, claw 0.5 mm.

####### Distribution.

Puget Sound to the southern California Bight; this is the first record from western Mexico.

####### Remarks.

Only a single female specimen was collected in this study. The specimen reported in this paper differs in some characteristics from the holotype of *Pycnogonum rickettsi*. However, we think it may be premature to describe this specimen as a new species because our specimen is a female and that described by Schmitt (1934) is a male.

## Supplementary Material

XML Treatment for Eurycyde bamberi

XML Treatment for Nymphopsis duodorsospinosa

XML Treatment for Callipallene californiensis

XML Treatment for Nymphon lituus

XML Treatment for Pycnogonum rickettsi
